# Proteomic analysis of SRA01/04 transfected with wild-type and mutant HSF4b identified from a Chinese congenital cataract family

**Published:** 2012-03-24

**Authors:** Aizhu Miao, Xinyan Zhang, Yongxiang Jiang, Yaohui Chen, Yanwen Fang, Hongfei Ye, Renyuan Chu, Yi Lu

**Affiliations:** 1 Department of Ophthalmology, Eye & ENT Hospital of Fudan University, Shanghai, P.R. China; 2 Department of Biochemistry and Molecular Biology, School of Life Sciences, Fudan University, Shanghai, P.R. China

## Abstract

**Purpose:**

Congenital cataracts account for about 10% of cases of childhood blindness. Heat shock transcription factor 4 (HSF4) is related with human autosomal dominant lamellar and Marner cataracts; a T→C transition at nucleotide 348 was found in a large Chinese cataract family. The aim of this study was to analyze the unique role of HSF4b and the mutation of HSF4b.

**Methods:**

The isobaric tags for relative and absolute quantification (iTRAQ), coupled with the two-dimensional liquid chromatography-tandem mass spectrometry (2D LC-MS/MS) technique, was used to identify and quantify differential proteomes in human lens epithelial cell lines SRA 01/04 expressing wild-type and mutant HSF4b.

**Results:**

A total of 104 unique proteins were identified from the human lens epithelial cell lines SRA 01/04. Apart from the proteins due to the effect of the pcDNA3.1 vector, the wild-type and mutant HSF4b led to 23 differentially expressed proteins, of which four were histone proteins and three were ribosomal proteins. The T→C transition at nucleotide 348 in HSF4b led to 18 differentially expressed proteins in SRA 01/04, among which serpin H1 precursor, heat shock protein beta-1, and stress-70 protein belong to heat shock protein families. The up- or down-regulated proteins were functionally analyzed using Ingenuity Pathways Analysis (IPA) to interpret the interaction network and predominant canonical pathways involved in these differentially expressed proteins.

**Conclusions:**

A multitude of differentially expressed proteins was found to be associated with HSF4b and a T→C transition at nucleotide 348 in *HSF4b*. The proteins interacted directly or indirectly with each other, and they may provide clues as to how HSF4b modulates protein expression in the lens epithelial cells of SRA 01/04. Although further investigation is required, the results may provide some new clues to the transcriptional mechanism of HSF4b and cataract formation.

## Introduction

A cataract is a visible opacity of the lens in the eye, which, when located on the visual axis, leads to vision loss [1]. Congenital cataracts account for about 10% of cases of childhood blindness [2], and approximately 50% of congenital cataracts are inherited [3]. Some transcription factor genes associated with congenital cataracts have been identified, including heat shock transcription factor 4 (HSF4).

In a previous study, the present authors found that HSF4, a highly conserved heat shock transcription factor, is related with human autosomal dominant lamellar and Marner cataracts [4]. In that study, four different missense mutations were identified within the HSF4 binding domain, among which a T→C transition at nucleotide 348 was found in all affected individuals in a Chinese family, but not in the unaffected members of the family or in 300 unrelated normal controls. A missense mutation of R74H was also identified as being related with autosomal dominant congenital total white cataracts in a Chinese family [5]. Apart from autosomal dominant cataracts, mutations in HSF4 may also result in autosomal recessive cataracts [6,7]. The role of HSF4 in hereditary cataracts in animals has also been demonstrated [8-10].

Inactivation of HSF4 in mice leads to early postnatal cataract formation with primary effects specific to terminal fiber cell differentiation [2]. HSF4 can bind to the heat shock element (HSE) and activate the transcription of downstream target genes. As heat shock genes are candidate targets of HSF4, it was shown that in HSF4 null mice that the expression of heat shock protein 27 (Hsp 27) in the lens reduced, while the expression of Hsp60, Hsp70, Hsp90, and Hsp110 increased. Interestingly, γ-crystallin expression was also markedly reduced in HSF4 null mice, leading to the suggestion that HSF4 directly binds and regulates the expression of γ-crystallin [11].

The target genes of *HSF4*, however, are yet unclear, and it remains unknown how mutations in *HSF4* lead to cataractogenesis. There are two isoforms of *HSF4* (HSF4a and HSF4b), which are derived from alternative RNA splicing events. HSF4a acts as a transcription inhibitor, while HSF4b, which contains 30 additional amino acids, acts as a transcription activator [12]. Only the expression of HSF4b can be detected in the lens [11].

In recent decades, site-directed mutagenesis has become an important technique in the study of protein-ligand or protein–protein interactions, which has enabled traditional methods to answer questions about structure-function relationships by allowing for the exchange of specific amino acid residues [13]. Meanwhile, cell transfection of mammalian cells has become a standard method of identifying mutant receptors. To determine the unique role of *HSF4b* and the T→C transition at nucleotide 348 in *HSF4b*, the wild-type and the mutant *HSF4b* were expressed in human lens epithelial cell lines SRA 01/04. The isobaric tags for relative and absolute quantification isobaric tags for relative and absolute quantification (iTRAQ), coupled with the two-dimensional liquid chromatography-tandem mass spectrometry (2D LC-MS/MS) technique, was used to identify and quantify differential proteomes in SRA 01/04 cell lines expressing wild-type and mutant *HSF4b*.

## Methods

### Reagents

iTRAQ reagents were purchased from Applied Biosystems Inc. (Foster City, CA). A NucleoBond Xtra Main kit was obtained from Macherey-Nagel (Düren, Germany), and a Lipofectamine^TM^ LTX kit was purchased from Invitrogen (Carlsbad, CA). Trypsin was obtained from Sigma Chemical Company (St. Louis, MO), and Dulbecco’s Modified Eagle Medium (DMEM) and fetal bovine serum (FBS) were purchased from Gibco (Invitrogen Corp., Mulgrave Victory, Australia).

### Human lens epithelial cell culture

Human lens epithelial cell lines SRA 01/04 were kindly provided by Dr. X.H. Shi of Shanghai Jiao Tong University School of Medicine, Shanghai Jiao Tong University School of Medicine, Shanghai, China. The cells were cultured in DMEM, supplemented with 10% FBS, in a humidified CO_2_ incubator. The medium was changed every other day until the time of collection.

### Site-directed mutagenesis, gene sequencing, and secondary structure prediction of HSF4b

The pcDNA3.1-HSF4b vector and pcDNA3.1 vector were kindly provided by Dr. X.H. Shi of Shanghai Jiao Tong University School of Medicine. Site-directed mutagenesis (T→C transition at nucleotide 348 in *HSF4b*) was performed using polymerase chain reaction (PCR) as follows: Forward (5′-GAG CAG CTA CC G GAG CGC GTG CGG CGC AAG-3′) and reverse (5′-GGT AGC TGC TCG CGG CCG CGC ACG AAG CTC G-3′) primers were designed. PCR amplification was performed in a total volume of 30 μl solution containing 30 ng of template DNA, 2 μl (10 μM) of each primer and 1 U of Pfu DNA polymerase. Each reaction was incubated for 5 min at 95 °C and 45 s at 95 °C, denatured at 55 °C for 30 s, and extended at 68 °C at a rate of 2 min/1 kb. After 30 cycles of amplification, PCR products were subjected to DpnI digestion to remove plasmid template DNA. The mutated plasmid was transformed into competent *E. coli* DH5α cells and then plated on LB-ampicillin plates. Positive clones were selected and cultured at 37 °C in LB medium. The accuracy of the mutation was confirmed by DNA sequencing. The secondary structure prediction of the HSF4b protein was performed using PepTool Lite software (Biotools Inc., Edmonton, Alberta), and HSF4b sequence alignments in different species were analyzed.

### Cell transfection

Wild-type pcDNA3.1-HSF4b, mutant pcDNA3.1-HSF4b, and empty vector pcDNA3.1 plasmid were extracted and purified using the NucleoBond Xtra Main kit according to the manufacturer’s instructions. Human lens epithelial cells SRA 01/04 were passaged at a density of 10^6^ cells/ml and cultured in 100 cm^2^ plates. pcDNA3.1-HSF4b, mutant pcDNA3.1-HSF4b and, empty vector pcDNA3.1 plasmid were transfected into SRA 01/04 under the same conditions when the cells adhered to the plates 8 h after passage using the Lipofectamine^™^ LTX kit. A fourth group of SRA 01/04 was also cultured without transfection under the same conditions.

### Protein preparation and iTRAQ labeling

Forty-eight hours after transfection, three plates (100 cm^2^) of cells from each group were collected for analysis. Proteins from each group were derived as follows: 100 μl Tris-buffered saline (0.5M, pH 7.4) was added to the collected cells. The mixture was subjected to a cycling program of 5 s ultrasonication and 10 s pause for 2min. Next, 400 μl solution (7 M urea, 2 M thiourea) was added, and mixed thoroughly for 30 min at 4 °C. The homogenates were centrifuged at 15,000× g for 45 min, and the supernatants were the required proteins. The total protein amount was determined using a standard Bradford protein assay.

iTRAQ labeling was done according to the kit protocol. A total of 100 μg of protein from each sample, in a volume of 20 μl dissolution buffer, was reduced with 1 μl of the denaturant and vortexed at 60 °C for 1 h to mix. Cysteine sulfhydryls were blocked by the addition of 1 μl cysteine-blocking reagent at room temperature for 10 min. Proteins were digested with 10 μl reconstituted trypsin and incubated at 37 °C overnight (12–16 h). Sample tubes were then spun to bring the sample digest to the bottom of each tube. Ethanol (70 μl) was added to each iTRAQ reagent, and then transferred to the appropriate sample tube. Peptides derived from cells transfected with empty vector pcDNA3.1 plasmid were labeled with iTRAQ tag 114, while peptides derived from cells without transfection, cells transfected with wild-type pcDNA3.1-HSF4b and cells transfected with mutant pcDNA3.1-HSF4b were labeled with tags 115, 116, and 117, respectively. Following incubation for 1 h at room temperature, the three tagged samples were combined in a fresh tube. The sample mixture was cleaned up using cation exchange chromatography for LC-MS/MS analysis.

### LC-MS/MS analysis

**Figure 1:**
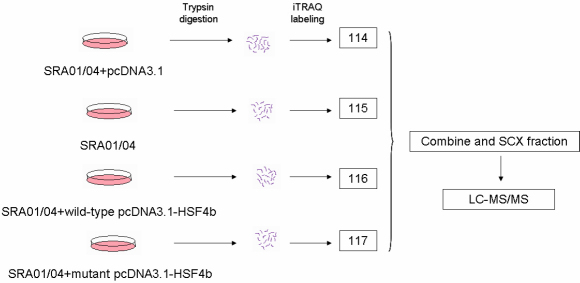
iTRAQ-based protein expression profiling.

A Shimadzu LC-20AD series HPLC system (Shimadzu; Kyoto, Japan) coupled to a QSTAR XL mass spectrometer (Applied Biosystems) via an electrospray ionisation (ESI) source was used for analysis. Peptide separation was performed by strong cation exchange (SCX) chromatography using a polysulfethyl column (2.1*100 mm, 5 μm, 300 Å; The Nest Group, Southborough, MA). The combined peptide mixture was diluted with the loading buffer (10 mM KH_2_PO_4_ in 25% acetonitrile [CAN], pH 2.6) and loaded onto the column. Buffer A was identical in composition to the loading buffer, and buffer B was buffer A containing 350 mM KCl. Separation was performed using a linear binary gradient of 0%–80% buffer B in buffer A at a flow rate of 200 μl/min for 60 min. A total of 20 SCX fractions were collected along the gradient. These fractions were dried down by the rotary vacuum concentrator, dissolved in buffer C (5% ACN, 0.1% formic acid [FA]), and analyzed on a QSTAR XL system (Applied Biosystems) interfaced with a 20AD HPLC system (Shimadzu). Peptides were separated on a ZORBAX 300SB-C18 enrichment column (0.1 * 15 mm, 5 µm, 300 Å; Microm, Auburn, CA). The HPLC gradient was 5%–35% buffer D (95% ACN, 0.1% FA) in buffer C at a flow rate of 0.3 μl/min for 70 min. For MS/MS analysis, survey scans were acquired from m/z 400 to 1800 with up to four precursors selected for MS/MS from m/z 100 to 2000 using dynamic exclusion. Each SCX fraction was analyzed in duplicate. The experimental protocol is illustrated in Figure 1.

### Data analysis

The MS/MS spectra were processed by a thorough search against the International Protein Index (IPI) database (version 3.45, HUMAN) using the Paragon and ProGroup Algorithm (Applied Biosystems). ProteinPilot Software (version 3.0, revision 114732; Applied Biosystems) was used to identify peptides and proteins and quantify differentially expressed proteins. The following parameters were set in the search: trypsin as enzyme, fixed modification of methylmethanethiosulfate-labeled cysteine, iTRAQ as sample type, no special factors, biologic modifications, and thorough identification search. Proteins that had at least one peptide with >95% confidence were considered for further statistical analysis. The quantification of proteins was performed using an averaging ratio, which was expressed as an error factor, EF=10^(95% confidence interval)^. An EF<2 was set for the quantification quality to be satisfied. To designate significant changes in protein expression, fold-changes >1.2 or <0.8 were set as cut-off values. The molecular functions of the unique proteins identified in the study were classified using IPA (Ingenuity Systems, Mountain View, CA).

## Results

### DNA sequencing and secondary structure prediction of Hsf4b

**Figure 2:**
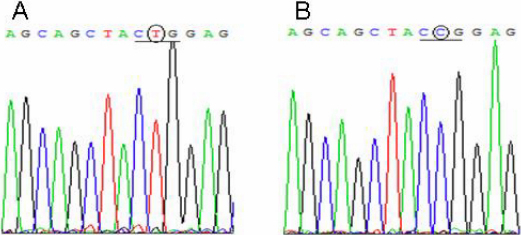
Sequence analysis of the wild-type and mutant *Hsf4b*. The data show the sequences of representative clones of wild-type (**A**) and mutant (**B**) *Hsf4b* and a T→C transition in the gene segment.

**Figure 3:**
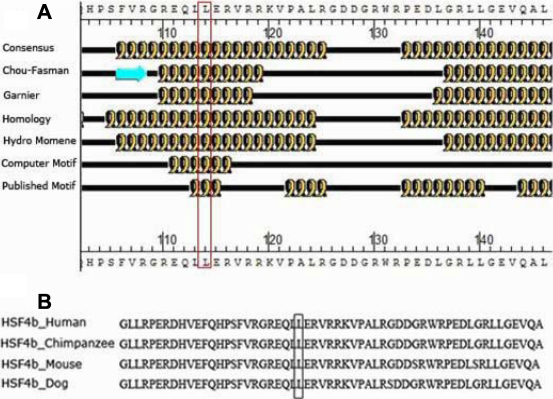
Secondary structure of HSF4b predicted by PepTool Lite software. **A**: Leu114Pro substitution was predicted to be located within a predicted α-helix of HSF4b. **B**: Alignments of HSF4b homologous sequences in different species.

Wild-type and mutant *HSF4b* were confirmed by DNA sequencing. The presence of the mutation (a T→C transition at nucleotide 348) in *HSF4* is shown in Figure 2. This mutation results in a Leu114Pro substitution in HSF4b. PepTool Lite software (Biotools Inc., Edmonton, Alberta, Canada) showed this mutation located within a predicted α-helix of HSF4b (Figure 3A) and alignments of HSF4b-homologous sequences in different species (human, chimpanzee, mouse and dog) showed that the mutation belonged to a conserved functional domain (Figure 3B).

### Identification of differentially expressed proteins

A total of 104 unique proteins were identified from four cell groups (cells transfected with empty vector pcDNA3.1 plasmid: 114; cells without transfection: 115; cells transfected with wild-type pcDNA3.1-HSF4b:116; and cells transfected with mutant pcDNA3.1-HSF4b: 117) according to the cut-off criteria (unused ProtScore >1.3 with at least one peptide with 95% confidence per repetition; Table 1). All identified proteins are presented in Appendix 1.

**Table 1:** Summary Statistics of proteomic result of International Protein Index data.

**Unused (confidence) cutoff**	**Protein detected**	**Protein before grouping**	**Distinct peptides**	**Spectra identified**	**% Total spectra**
>2.0 (99)	68	317	938	2177	7.8
>1.3 (95)	104	521	1102	2370	8.5
>0.47 (66)	122	610	1208	2485	8.9
Cutoff Applied:>1.3 (95%)^a^	104	521	1102	2370	8.5

^a^This row shows the unused Protscore threshold applied in this paper and the corresponding statistical result.

To designate significant changes in protein expression, fold changes >1.2 or <0.8 were set as cut-off values. Cell group 117 (cells transfected with mutant pcDNA3.1-HSF4b) had eight significant upregulated proteins and four significant down-regulated proteins, while cell group 116 (cells transfected with wild-type pcDNA3.1-HSF4b) had nine significant upregulated proteins and five significant down-regulated proteins compared with cell group 114 (cells transfected with empty vector pcDNA3.1 plasmid; Table 2). Cell group 114 had 11 significant upregulated proteins and 5 significant down-regulated proteins compared with cell group 115 (cells without transfection; Table 3). Cell group 117 had 11 significant upregulated proteins and 7 significant down-regulated proteins compared with cell group 116 (Table 4). Apart from Isoform 2 of Vinculin significantly upregulated and protein S100-A13 significantly down-regulated in both 117:114 and 116:114, there were 24 significant changed proteins (shown in Table 2). The 16 proteins in Table 3 were due to the effect of the pcDNA3.1 vector on SRA 01/04, among which ribosomal protein L1 was also found upregulated in Table 2. Therefore, 23 proteins were differentially regulated by wild-type and mutant HSF4b according to Table 2 and Table 3.

**Table 2:** Differentially expressed proteins in cells transfected with mutant pcDNA3.1-HSF4b (Group 117) and cells transfected with wild-type pcDNA3.1-HSF4b (Group 116) compared with cells transfected with empty vector pcDNA3.1 plasmid (Group 114).

**Significant upregulated proteins**				
**N**	**117:114**	**Protein name**	**116:114**	**Protein name**
1	2.004401	Serpin H1 precursor	1.757005	ALB protein
2	1.608196	Vesicle-fusing ATPase	1.688435	Malate dehydrogenase, mitochondrial precursor
3	1.563932	Ribosomal protein L7a	1.383886	Isoform 2 of Vinculin
4	1.313923	Isoform 2 of Vinculin	1.309574	Ribosomal protein L1
5	1.265481	Ubiquitin carboxyl-terminal hydrolase isozyme L1	1.299549	Isoform 1 of Kinectin
6	1.261812	Profilin-1	1.289466	H2A histone family, member J
7	1.21838	proteasome activator subunit 1 isoform 2	1.256752	ATP synthase subunit alpha, mitochondrial precursor
8	1.211856	40S ribosomal protein S20	1.214084	Isoform 2 of Somatotropin precursor
9			1.205054	Stress-70 protein, mitochondrial precursor
**Significant down-regulated proteins**				
1	0.799037	Protein S100-A13	0.797662	Protein DJ-1
2	0.796461	protein kinase C substrate 80K-H isoform 2	0.757478	Protein S100-A13
3	0.785344	Histone H4	0.750524	Histone H3.3
4	0.767087	Histone H1.5	0.734246	40S ribosomal protein S2
5			0.726876	Heat shock protein beta-1

**Table 3:** Differentially expressed proteins in cells transfected with empty vector pcDNA3.1 plasmid (Group 114) compared with cells without transfection (Group 115).

**Significant upregulated proteins**		
**N**	**114:115**	**Protein Name**
1	1.637794	plastin 3
2	1.519839	Protein S100-A13
3	1.35828	protein kinase C substrate 80K-H isoform 2
4	1.32791	Protein disulfide-isomerase A3 precursor
5	1.283924	Transgelin-2
6	1.274584	HSPA5 protein
7	1.265127	Histone H4
8	1.256839	Ribosomal protein L1
9	1.223014	40S ribosomal protein S15
10	1.221904	Isoform 1 of Protein disulfide-isomerase A6 precursor
11	1.220482	Calmodulin
**Significant down-regulated proteins**		
**N**	**114:115**	**Protein Name**
1	0.780324	40S ribosomal protein S20
2	0.778381	Keratin, type I cytoskeletal 18
3	0.727208	Beta-actin-like protein 2
4	0.625087	Isoform 2 of Vinculin
5	0.607064	Vesicle-fusing ATPase

**Table 4:** Differentially expressed proteins in cells transfected with mutant pcDNA3.1-HSF4b (Group 117) compared with cells transfected with wild-type pcDNA3.1-HSF4b (Group 116).

**Significant upregulated proteins**		
**N**	**117:116**	**Protein Name**
1	3.212416	Ribosomal protein L7a
2	1.655658	Serpin H1 precursor
3	1.402194	UPF0556 protein C19orf10 precursor
4	1.385797	Ubiquitin carboxyl-terminal hydrolase isozyme L1
5	1.37898	Heat shock protein beta-1
6	1.347777	40S ribosomal protein S2
7	1.290956	Histone H3.3
8	1.288081	Protein DJ-1
9	1.270944	Profilin-1
10	1.220242	Peptidyl-prolyl cis-trans isomerase A
11	1.205148	14–3-3 protein
**Significant down-regulated proteins**		
**N**	**117:116**	**Protein Name**
1	0.788502	H2A histone family, member J
2	0.743393	Stress-70 protein, mitochondrial precursor
3	0.72295	ATP synthase subunit alpha, mitochondrial precursor
4	0.663274	Isoform 1 of Kinectin
5	0.631487	Ribosomal protein L1
6	0.622737	Malate dehydrogenase, mitochondrial precursor
7	0.617919	ALB protein

**Figure 4:**
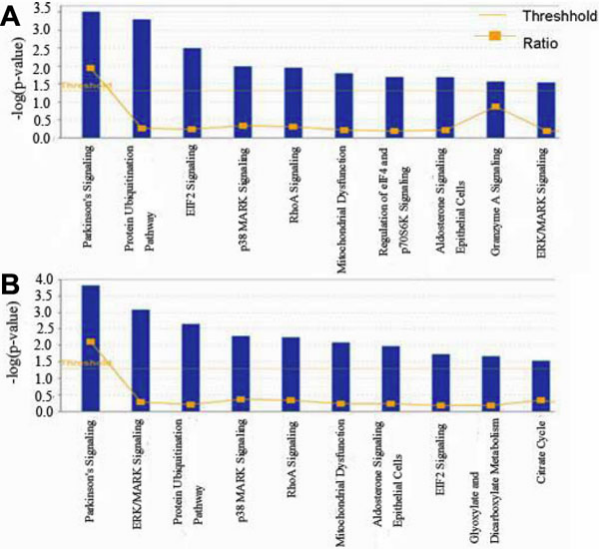
Predominant canonical pathways of differentially expressed proteins identified by ingenuity pathway analysis (IPA). **A**: Differentially expressed proteins caused by wild-type and mutant HSF4b. **B**: Differentially expressed proteins caused by T→C transition at nucleotide 348 in *HSF4b*.

**Figure 5:**
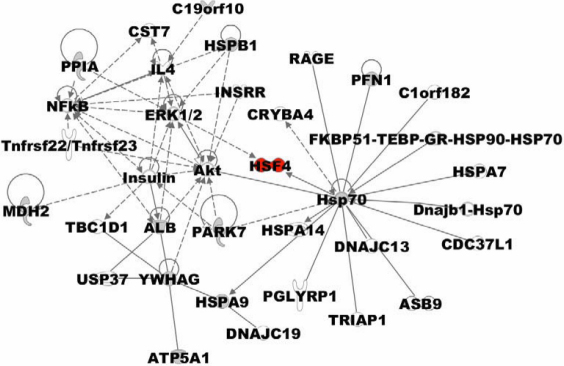
Analysis of up- and down- regulated proteins by T→C transition at nucleotide 348 in *HSF4b* using ingenuity pathway analysis. Proteins highlighted in gray shading were identified in the LC-MS/MS analysis. Proteins highlighted in clear were not identified in the LC-MS/MS analysis but were linked to the identified protein by examination of the ingenuity pathway analysis curated database describing protein:protein interactions. Lines connecting the molecules indicate molecular relationships. Dashed lines indicate indirect interactions and solid lines indicate direct interactions.

In addition, the up- and down-regulated proteins in Table 4 were in relevance to the effect of the T→C transition at nucleotide 348 in HSF4b. All proteins in Table 2 (except ribosomal protein L1) and the proteins in Table 4 were functionally analyzed using IPA separately to interpret the predominant canonical pathways and interaction network involved in these differentially expressed proteins. The predominant canonical pathways identified from the IPA library are shown in Figure 4. A network was generated by uploading the proteins in Table 4, as shown in Figure 5.

## Discussion

The current study explored the proteome expression of human lens epithelial cell lines (SRA 01/04) to demonstrate the effect of *HSF4b* and the T→C transition at nucleotide 348 in HSF4b on SRA 01/04. It is well known that HSF4 is necessary for the expression of heat shock genes, and that it also regulates non-classical heat shock genes in lens cells [14]. HSF4 was identified as a novel cataractogenic transcription regulatory factor whose mutation was closely associated with congenital cataracts in humans and animals. In humans, missense mutations in the *HSF4* gene cause cataracts. *HSF4*-knockout mice have been demonstrated to undergo impaired lens development, and exhibit abnormalities in lens fiber cell differentiation and cataract formation [2,11,15]. However, the role of HSF4b, an isoform of HSF4, has only recently been reported [16-18], and the transcriptional mechanism of *HSF4b* remains unclear. Mutations in *HSF4* were first reported to be related with human autosomal dominant lamellar and Marner cataracts in 2002 [4], and the T→C transition at nucleotide 348 in HSF4 was discovered in the affected individuals of a large Chinese congenital cataract family. However, the transcriptional activity of *HSF4b* remains unclear. The secondary structure prediction of the HSF4b protein by PepTool Lite software (Biotools Inc.) showed the Leu114Pro substitution in HSF4b that resulted from the T→C transition at nucleotide 348 in HSF4b located within a predicted α-helix of HSF4b (Figure 3A). This mutation might interrupt the α-helix structure of HSF4b. Alignments of HSF4b homologous sequences in different species (human, chimpanzee, mouse, and dog) showed that the mutation belonged to a conserved functional domain. In the present study, the authors conducted experiments with SRA01/04 cells transfected with wild-type and mutant HSF4b and explored the proteome expression of the cell proteins using the iTRAQ quantitative proteome method.

The study found 24 significant changed proteins in 117:114 (cells transfected with mutant pcDNA3.1-HSF4b: cells transfected with empty vector pcDNA3.1 plasmid) and 116:114 (cells transfected with wild-type pcDNA3.1-HSF4b: cells transfected with empty vector pcDNA3.1 plasmid). Apart from the proteins due to the effect of the pcDNA3.1 vector, the wild-type and mutant HSF4b led to 23 differentially expressed proteins in SRA01/04. Similarly, 18 unique up- or down-regulated proteins were identified in 117:116 (cells transfected with mutant pcDNA3.1-HSF4b: cells transfected with wild-type pcDNA3.1-HSF4b), which indicated that the T→C transition at nucleotide 348 in HSF4b led to 18 differentially expressed proteins in SRA 01/04. It was noted that in the 23 significant up- or down-regulated expressed proteins, four were histone proteins and three were ribosomal proteins.

Fujimoto et al. [14] found that HSF4 has an effect on histone. It binds to various genomic regions, and HSF4 binding induces the demethylation of histone H3K9 on the binding regions. Ribosomal proteins modulate protein synthesis. Zhang et al. [19] found that ribosomal protein L7a exhibited decreased expression in age-related cataracts compared with normal human lenses. HSF4b binds to HSE during the G_1_ phase of the cell cycle and stimulates the expression of heat shock proteins [17].

The present study showed that these 18 differentially expressed proteins were regulated directly or indirectly by mutant HSF4b, among which serpin H1 precursor (also known as 47 kDa heat shock protein), heat shock protein beta-1, and stress-70 protein (also known as heat shock 70 kDa protein 9) belong to heat shock protein families. The heat shock proteins were related with protein folding and proteolysis.

Heat shock proteins, functioning as molecular chaperones, are critical for maintaining proper protein folding and the degradation of unfolded protein [20]. The normal ocular lens is a transparent tissue. Cataracts occur when the lens becomes opaque, and they are associated with the aggregation of partially unfolded or damaged proteins in the lens [21]. Heat shock protein beta-1 (also known as Hsp27) has been found to function as a molecular chaperone suppressing the aggregation of specific client polypeptides. Transgenic mice overexpressing Hsp27 have been found to be strongly protected against myocardial infarction and cerebral ischemia [22,23]. A decrease in the level of expression of Hsp27 impairs growth and cytoskeletal organization [24]. Heat shock proteins are related with cataracts, and Hsp27 has been found to be a potent protective factor and even a therapeutic target for cataracts [25]. It has been found that the expression of heat shock protein 70 is significant in diabetic human lens epithelial cells, which implies that it may play a critical role in the development and formation of human diabetic cataracts [26]. It has also been found that HSF4b expression in cells leads to the enhanced synthesis of inducible heat shock protein 70 [17].

The differentially expressed proteins by wild-type and mutant HSF4b were analyzed in the canonical pathways identified from the IPA library to investigate the relationship between those proteins and various biologic pathways. The canonical pathways that were identified from the IPA library based on their significance to the data set are shown in Figure 4. The protein ubiquitination pathway and extracellular signal-regulated kinase/mitogen-activated protein kinase (ERK/MAPK) signaling were included. The ubiquitin proteasome system is involved in differentiation and pathological processes in the eye lens. The expression of the ubiquitin proteasome system has been found to be upregulated during the differentiation phase of the lens fiber cells [27,28]. The lens ubiquitin proteasome system responds to oxidative stress with increased activity and degrades damaged proteins in a highly selective way [29,30]. The results of the current study are also consistent with those of Tu et al. [16], who determined that HSF4b is a direct target of mitogen-activated protein (MAP) kinase extracellular signal-related kinase (ERK) and that phosphorylation of HSF4b by ERK leads to the binding of HSF4b to DNA.

In conclusion, in the current study, a multitude of differentially expressed proteins was found to be associated with HSF4b and a T→C transition at nucleotide 348 in HSF4b. The proteins interacted directly or indirectly with each other, and they may provide clues as to how HSF4b modulates protein expression in the lens epithelial cells of SRA 01/04. Although further investigation is needed to understand the transcription activity of HSF4b, the results of the current study may provide some new clues to the transcription mechanism of HSF4b. They may also offer new insight into the associations between the differentially expressed proteins and HSF4b. The networks among these differently expressed proteins may impair the differentiation of lens epithelial cells and result in cataract formation.

## Appendix 1. Total identified proteins.

To access the data, click or select the words “Appendix 1.” This will initiate the download of a compressed (pdf) archive that contains the file.
